# Lignans from the bark of *Eucommia ulmoides* inhibited Ang II-stimulated extracellular matrix biosynthesis in mesangial cells

**DOI:** 10.1186/1749-8546-9-8

**Published:** 2014-02-14

**Authors:** Zhen-yu Li, Xiao-lan Deng, Wei-hua Huang, Ling Li, Hui Li, Xian Jing, Ying-ying Tian, Pei-yu Lv, Tian-lun Yang, Hong-hao Zhou, Dong-sheng Ouyang

**Affiliations:** 1 The Xiangya Hospital Central-South University, Changsha, Hunan 410078, China; 2 Institute of Clinical Pharmacology, Hunan Key Laboratory of Pharmacogenetics, Central South University, 110 Xiang-Ya Street, Changsha, Hunan 410078, China; 3 Haikou Hospital Affiliated to Xiangya Central-South University, Haikou, Hainan 570208, China; 4 Institute of Hepatobiliary Diseases of Wuhan University, Zhongnan Hospital of Wuhan University, Wuhan, Hubei 430071, China

## Abstract

**Background:**

Tree bark of *Eucommia ulmoides* Oliv., (commonly well-known as “*Du-zhong*” in China), has been used to treat hypertension, hypercholesterolemia, hyperglycemia, hepatic fibrosis and renal injury. This study aims to investigate the effects of lignans extracted from the bark of *Eucommia ulmoides* Oliv. on Ang II-induced proliferation and extracellular matrix biosynthesis in rat mesangial cells.

**Methods:**

Rat mesangial cells (RMCs) were cultured *in vitro* and divided into six groups (control, Ang II, losartan, and low, middle and high concentration lignans groups). RMC proliferation was measured by MTT assay. RT-qPCR and western blotting were used to detect mRNA and protein expression of collagen type I (Col I), collagen type III (Col III), collagen type IV (Col IV), fibronectin and aldose reductase (AR).

**Results:**

Cellular proliferation induced by Ang II was significantly suppressed by *Eucommia* lignans of different concentrations (*P* = 0.034, *P* < 0.001, and *P* < 0.001). Treatment of cells with Ang II increased Col I, Col III, Col IV, and fibronectin mRNA expression, which was observed at the protein level (*P <* 0.001, *P <* 0.001, *P* = 0.004, and *P* = 0.004, respectively). The increased mRNA expression and protein levels of Col I, Col III, Col IV, and fibronectin were diminished remarkably with by treatment *Eucommia* lignans, and elevated AR expression stimulated by Ang II was significantly inhibited by *Eucommia* lignans.

**Conclusions:**

*Eucommia* lignans (*Du-zhong*) inhibited Ang II-stimulated extracellular matrix biosynthesis in mesangial cells.

## Background

Hypertension is the second leading cause of end-stage renal disease [1], resulting in hypertensive nephropathy, which usually starts with glomerulosclerosis [2,3]. The pathophysiologic process is associated with structural changes to renal glomeruli due to mesangial cell (MC) proliferation and abnormal accumulation of extracellular matrix (ECM) [3,4]. Angiotensin II (Ang II), the main effective peptide in the rennin-angiotensin system (RAS) is considered a key mediator in the development of hypertensive glomerulosclerosis [3,5]. RAS regulates the proliferation of MCs and increases the production of ECM, mainly through the induction of glomerular hypertension, as well as non-hemodynamic effects, which include the production of reactive oxygen species, up-regulation of profibrotic growth factors, such as platelet-derived growth factor (PDGF), transforming growth factor-β1 (TGF-β1), tumor necrosis factor-α (TNF-α) and connective tissue growth factor, and macrophage activation and infiltration [3,6-8].

Aldose reductase (AR), a member of the aldo-ketoreductase super family, catalyzes the conversion of glucose to sorbitol dependent on NADPH in the first step of the polyol pathway [9]. AR can be activated by TGF-β1, oxidative stress and inflammation [9-12], to stimulate proliferation of MCs and deposition of ECM induced by TGF-β1 and PDGF [13-16]. An over-expression of AR was observed in the renal tissue of spontaneous hypertensive rats (SHR) in our previous study [12]. AR might be involved in the pathological process induced by Ang II in MCs.

Cortex Eucommiae (*Du-zhon*g), the dried bark of the *Eucommia ulmoides* Oliv. (Family: Eucommiaceae), has been served as a traditional tonic medicine and is thought to benefit the liver and kidney, strengthen tendons and bones, and prevent miscarriage [17]. The natural products that have been identified from *Du-zhong* include lignans, iridoids, flavonoids, polysaccharides, terpenes and protein [18], which possess various pharmacological effects, including antihypertensive, antioxidant, antimicrobial, and anti-inflammatory properties [9,18]. We previously confirmed that lignans were the effective fraction of *Du-zhong* for antihypertension [10,19]. Further study showed that both N-acetyl-β-D-glucosaminidase enzyme activity and the ratio of albumin to urinary creatinine decreased in spontaneous hypertensive rat (SHR)-treated with *Eucommia* lignans [12]. *Eucommia* lignans also inhibited the expression of collagen type III (Col III) in the glomerular basement membrane, and diminished the over-expression of AR in the kidney [11]. Accordingly, we hypothesized that lignans decreased the production of Col III by affecting AR expression and thus decreased damage to the glomerular structure [12].

This study aims to investigate the effects of lignans extracted from the bark of *Eucommia ulmoides* Oliv. On Ang II-induced proliferation and extracellular matrix biosynthesis in rat mesangial cells, and attempted to elucidate the mechanisms by which *Eucommia* lignans from *Du-zhong* protecting against hypertensive renal injury *in vitro*.

## Methods

### Materials and reagents

RPMI-1640 medium, newborn calf serum (NCS), RT-qPCR kits with Platinum® SYBR® Green qPCR Super Mix-UDG, and primers for collagen I (Col I), Col III, collagen type IV (Col IV), fibronectin and AR were purchased from Invitrogen (Carlsbad, CA, USA). Antibodies for Col I, Col III, Col IV, fibronectin and AR for western blotting were supplied by Abcam (Cambridge, England) and Santa Cruz Biotechnology Inc. (CA, USA). The Cell Titer 96® Aqueous One Solution Proliferation Assay for the 3-(4, 5-dimethylthiazol-2-yl)-2, 5-diphenyl tetrazolium bromide (MTT) method was provided by Promega (Madison, WI, USA). A Revert Aid First Strand cDNA Synthesis Kit was purchased from Thermo Scientific (Austin, TX, USA). Losartan was obtained commercially from the National Institute for Food and Drug Control (Beijing, China). Human Angiotensin II and other analytical grade reagents were purchased from Sigma-Aldrich (St. Louis, MO, USA).

*Eucommia* lignans were extracted at our own laboratory as described previously [12,19,20]. *E. ulmoides* were obtained from Changsha Medical Company (Hunan, China) in July 2009, and authenticated by Dr. Dong-Sheng Ouyang, one of the authors according to the methods described in the literature [21]. A voucher specimen of *Eucommia ulmoides* Oliv. (IBSC_0345347) was deposited at South China Botanical Garden Herbarium, Guangdong, China. Briefly, fresh *Eucommia ulmoides* Oliv. bark was cut into pieces and extracted with 60% ethanol purchased from Changsha Tianshun Chemical Co., Ltd (Hunan, China) at 70°C for 2 h (twice). The extract was subjected to macroporous resin supplied by Haiguang Chemical Industrial Company (Tianjin, China) and eluted with 80% ethanol after treatment with pure water as the eluent. The eluent was freeze-dried to powder and stored at 4°C. The lignans content in *Eucommia* lignans was 71%, as determined by spectrophotometry on a Beckman Coulter DU 640 spectrophotometer (Beckman Coulter, Inc. USA) at 277 nm, with pinoresinoldiglucoside used as the control which was supplied by college of chemistry and chemical engineering in Central South University (Hunan, China).

### Cell culture

RMCs (HBZY-1 cells) were purchased from China Center for Type Culture Collection (Wuhan, China). After recovery, RMCs were cultured in RPMI-1640 medium supplemented with 10% NCS at 37°C in a humidified atmosphere of 5% CO_2_ in air.

### MTT assay

RMCs were added into the wells of a 96-well plate at a density of 3000 cells per well and cultured in RPMI-1640 medium containing 10% NCS. All incubations were performed in RPMI-1640 containing 1% NCS when they grew to 60% confluence. The study included two parts: (1) Control group, *Eucommia* lignans groups (10, 20, 30, 40, 50, 60, 70, 80, or 90 mg/L *Eucommia* lignans); and (2) Control group, Ang II group (10 nM Ang II), Losartan group (10 nM Ang II with 20 μM Losartan), *Eucommia* lignans groups (10 nM Ang II with 20, 40 and 80 mg/L *Eucommia* lignans). After 48 h, the viability of RMCs was measuredby MTT method. Then, 20 μL cell Titer 96® Aqueous One Solution Reagent was added to the medium in each well, and the absorbance of solubilized blue formazan was recorded by a microplate reader (Molecular Devices, Spectra MAX. 250, USA) at 490 nm after 1 h at 37°C in a humidified 5% CO_2_ atmosphere.

### Reverse transcription real-time quantitative PCR (RT-qPCR) assay

RMCs were assigned to six groups: Control group, Ang II group (10 nM Ang II), Losartan group (10 nM Ang II with 20 μM Losartan), and *Eucommia* lignans groups (10 nM Ang II with 20, 40 or 80 mg/L *Eucommia* lignans), in a 6-well plate, and cultured in RPMI-1640 medium containing 10% NCS for 48 h. Total RNA from RMCs was extracted by Trizol® reagent (Invitrogen, Carlsbad, CA, USA) and the concentration was determined by spectrophotometry at 260 and 280 nm. A reverted aid cDNA synthesis kit was used to perform the synthesis of first strand cDNA from total RNA templates. Real-time qPCR was performed by Platinum® SYBR® Green qPCR Super Mix-UDG following the manufacturer’s instructions. The gene-specific primers are listed in Table 1. The data were quantitatively analyzed by Stratagene Mx3000p Real-time PCR (Santa Clara, CA, USA). The glyceraldehyde phosphate dehydrogenase (GAPDH) gene was used as the internal control.

**Table 1:** Information on the primers used for real-time PCR

**Gene**	**Primers**	**Product size (bp)**
AR	F: TCCCAGGATCAAGGAAATTG	202
	R: ACAACAGGAACTGGAGGGTG	
FN	F: AACGGCCCTGGTTTGTACC	285
	R: CTCCAACATATAGCCACCAGTC	
Col I	F: GAGAGAGCATGACCGATGGA	251
	R: CGTGCTGTAGGTGAATCGAC	
Col III	F: GGCTGCACTAAACACACTGG	229
	R: TGGTTGACGAGATTAAAGCAAG	
Col IV	F: TGTCAGCAATTAGGCAGGTC	205
	R: CACCATGTTTCGGAATGGTT	
GAPDH	F: CAAGTTCAACGGCACAGTCAAG	123
	R: ACATACTCAGCACCAGCATCAC	

### Western blotting

Total protein was extracted from RMCs with radio immunoprecipitation assay lysis buffer consisting of 10 mM sodium phosphate (pH 7–8), 150 mM NaCl, 0.1% SDS, 0.5% sodium deoxycholate and 1% Triton X 100 after a 48-h culture under the conditions described above, and the protein concentration was determined using a bicinchoninic acid assay kit (Sigma-Aldrich, USA). A total of 40 μg of total protein was separated on a 10% sodium dodecyl sulfate polyacrylamide gel (Invitrogen, Carlsbad, CA, USA) and transferred onto a polyvinylidenefluoride membrane (Millipore, USA). The membrane was blocked with 5% skim milk solution in 0.1% tris-buffered saline Tween-20 (TBST) (Shanghai Bioscience Co. Ltd, China) over night. Subsequently, one of the primary antibodies (rabbit polyclonal for Col I, mouse monoclonal [FH-7A] for Col III, rabbit polyclonal for Col IV, mouse monoclonal [IST-9] for fibronectin, goat polyclonal for AR, and rabbit polyclonal for GAPDH) was added for hybridization before being incubated with the specific secondary antibody after washing membranes with TBST (Shanghai Bioscience Co. Ltd, China) three times. Protein bands were determined by the enhanced chemiluminescence western blotting detection system. GAPDH was used as an internal standard for data normalization.

### Statistical analysis

Data were shown as mean ± standard deviation (SD) and were analyzed with SPSS 17.0 software (SPSS Inc., USA). A *P* value less than 0.05 was considered statistically significant. Significant differences between multiple groups were analyzed by one-way analysis of variance (ANOVA) followed by a Dunnett’s post-*hoc* test.

## Results

### Effects of *Eucommia* lignans on RMC growth

**Figure 1:**
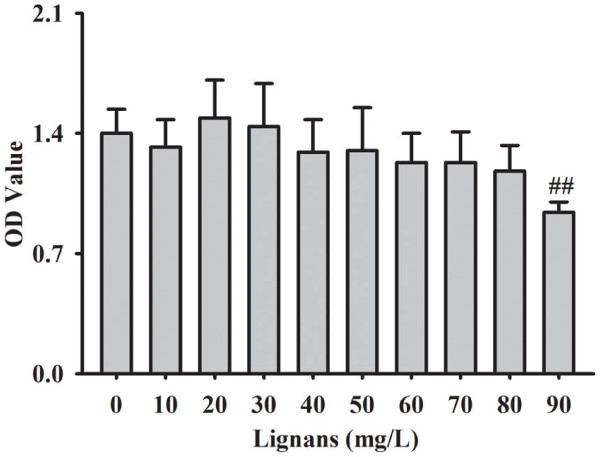
**Effects of *Eucommia *lignans on RMCs growth.** RMCs were treated with various concentrations of *Eucommia* lignans (10, 20, 30, 40, 50, 60, 70, 80, or 90 mg/L) for 48 h, and cell viability was assessed by MTT method. Results were given in mean ± SD (n = 6). ^##^*P* < 0.01 *vs.* the control group.

In comparison with the control, there was no significant change in the number of cells treated with *Eucommia* lignans in the 10, 20, 30, 40, 50, 60, 70 and 80 mg/L groups (*P* = 0.983, 0.986, 1.000, 0.900, 0.920, 0.515, 0.515, and 0.249, respectively). However, cellular viability decreased markedly in the group incubated with 90 mg/L *Eucommia* lignans (*P* = 0.001) (Figure 1). Therefore, the incubated concentrations of *Eucommia* lignans for the following experiments were 20, 40 and 80 mg/L.

### Inhibition of *Eucommia* lignans on Ang II-induced RMC proliferation

**Figure 2:**
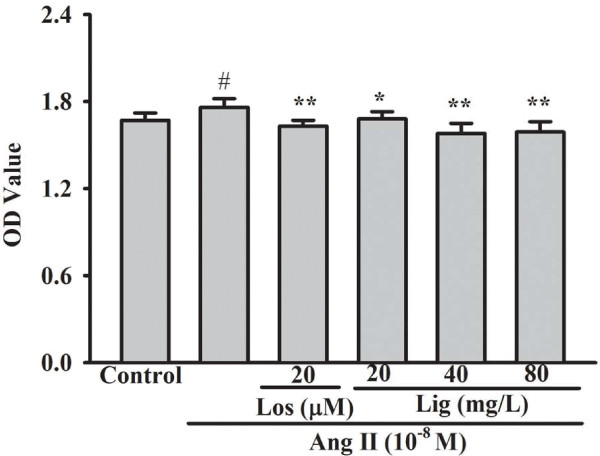
**Inhibitory effects of *Eucommia *lignans on Ang II-induced RMCs proliferation.** All values represented mean ± SD (n = 6). Los: losartan; Lig: lignans. ^#^*P* < 0.05 *vs.* the control group, ^*^P < 0.05, ^**^P < 0.01 *vs.* Ang II group.

The Ang II receptor blocker, losartan (20 μM), significantly decreased the proliferation of RMCs induced by Ang II (*P* = 0.004). The inhibitory effects were also observed in the different *Eucommia* lignans-treated groups (*P* = 0.034, *P* < 0.001, and *P* < 0.001) (Figure 2).

### Reduction of *Eucommia* lignans on Ang II-induced ECM biosynthesis in RMCs

**Figure 3:**
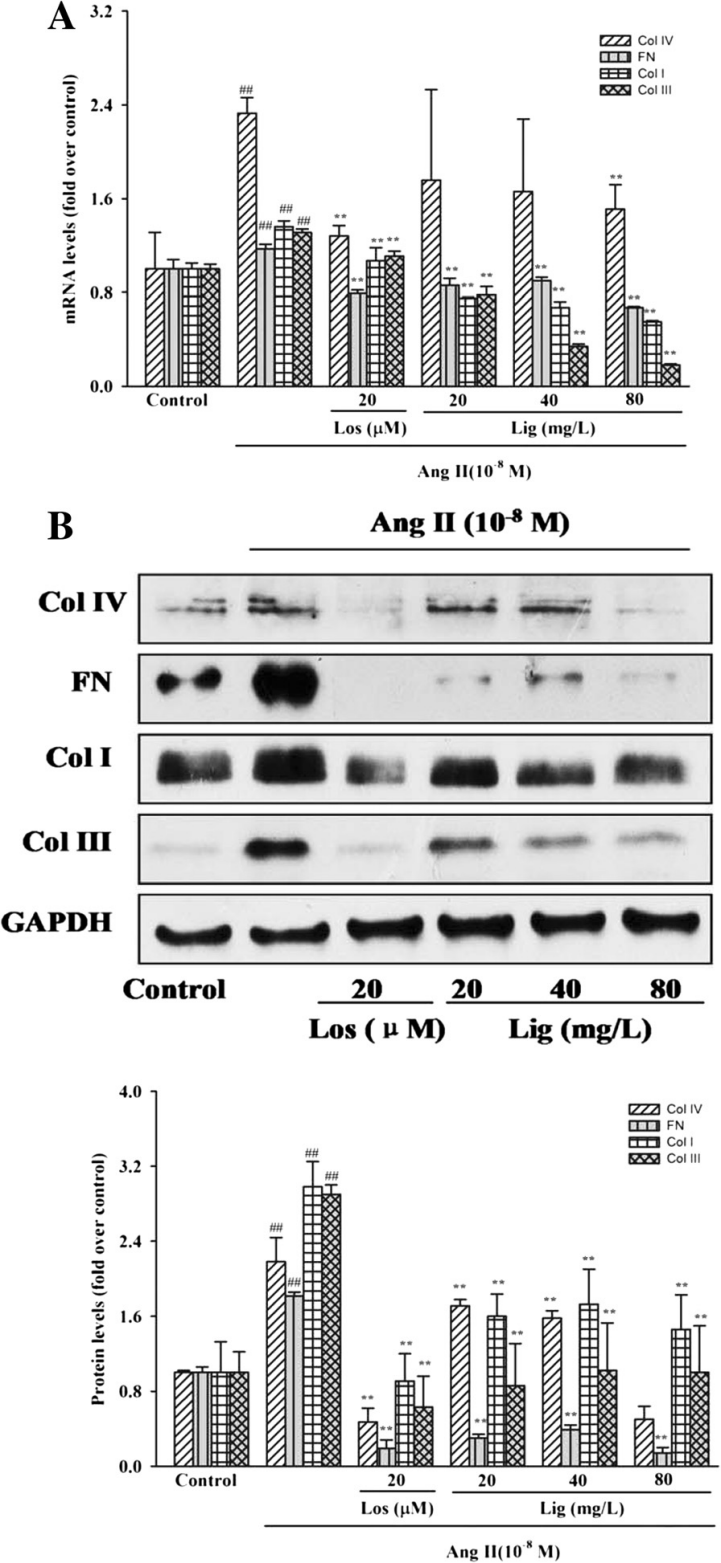
**Inhibitory effects of *Eucommia *lignans on Ang II-induced ECM biosynthesis in RMCs. (A)** depression of *Eucommia* lignans on Ang II-increased mRNA expression levels; **(B)** inhibition of *Eucommia* lignans on Ang II-stimulated protein expression levels. *RT-qPCR* and western blot was used to detect the mRNA and protein expressions separately. GAPDH was used as an internal loading control gene. Both mRNA and protein relative expression levels were expressed as folds of control. All values were expressed as mean ± SD (n = 3). Los: losartan; Lig: lignans. ^##^*P* < 0.01 *vs.* the control group, ^**^P < 0.01 *vs.* Ang II group.

The changes in Col I, Col III, Col IV and fibronectin production are shown in Figure 3. mRNA and protein expression increased with Ang II stimulation. All of the increased expression levels induced by Ang II could be attenuated by losartan treatment (Col IV mRNA, *P* = 0.007; others, *P* < 0.001). Furthermore, *Eucommia* lignans also significantly diminished their ascended expression (Col I protein of 20 mg/L, *P* = 0.001; Col I protein of 40 mg/L, *P* = 0.002; Col IV mRNA of 80 mg/L, *P* = 0.031; others, *P* < 0.001), although decreases of the Col IV mRNA level of the low and middle concentration lignans groups did not reach a statistically significant difference (*P* = 0.084 and *P* = 0.134). *Eucommia* lignans could suppress Ang II-stimulated biosynthesis of ECM in RMCs (Figure 3B).

### Block of *Eucommia* lignans on Ang II-induced AR expression in RMCs

**Figure 4:**
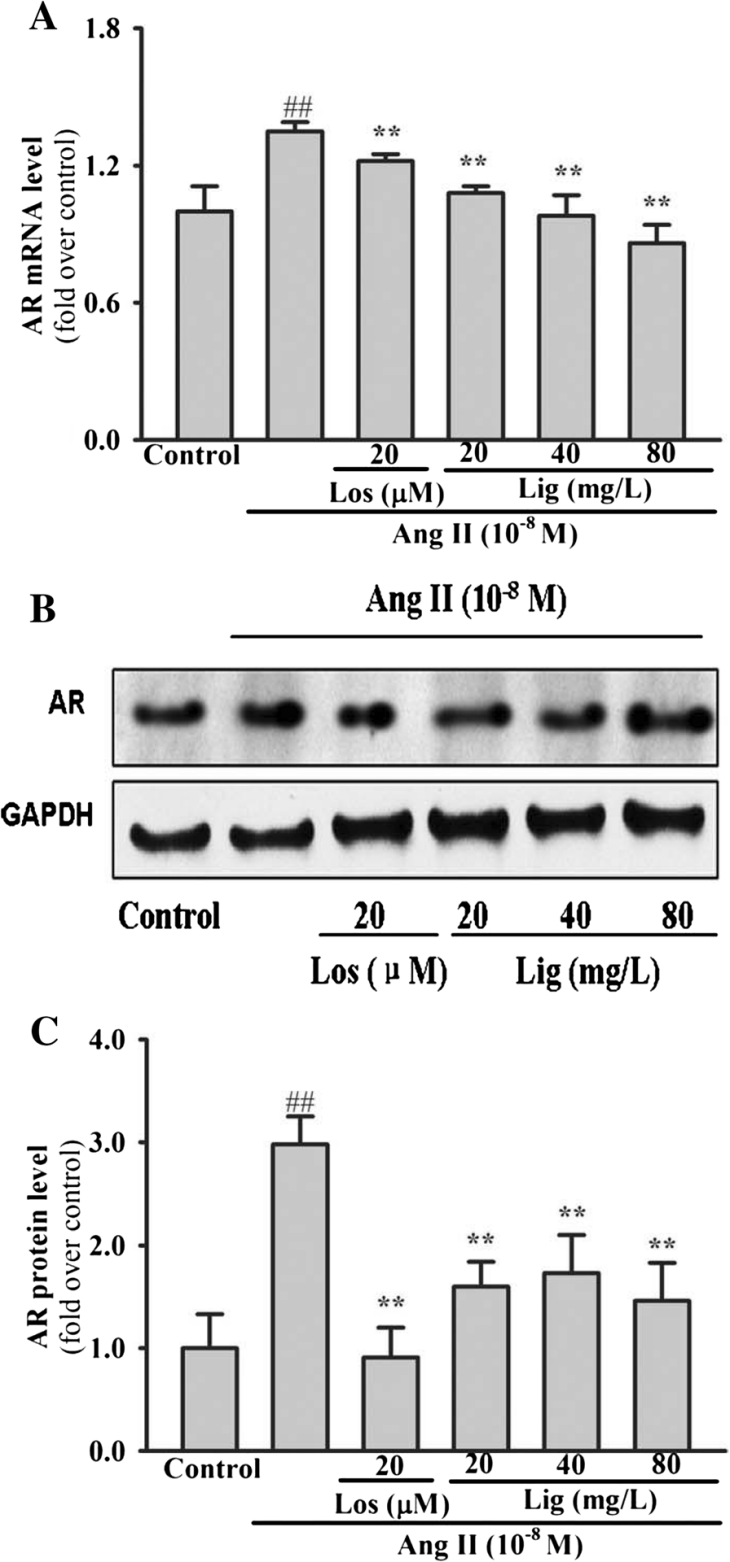
**Inhibitory effects of *Eucommia *lignans on Ang II-induced AR expression in RMCs. (A)** Inhibition of *Eucommia* lignans on Ang II-increased AR mRNA levels; **(B and C)** depression of Eucommia lignans on Ang II-stimulated AR protein expression. Cells were incubated as described in Methods. *RT-qPCR*and Western blotting was used to detect the mRNA and protein expressions. GAPDH was selected as an internal standard gene. AR relative expression levels were indicated as folds of control. All results were shown in mean ± SD (n = 3). Los: losartan; Lig: lignans. ^##^*P* < 0.01 *vs.* the control group, ^**^P < 0.01 *vs.* Ang II group.

The mechanisms of *Eucommia* lignans inhibitory effects were tentatively elucidated from data of our previous animal experiments [22]. Both mRNA and protein expression of AR were effectively enhanced by Ang II (*P* < 0.001) (Figure 4). Losartan and *Eucommia* lignans clearly attenuated all expression stimulated by Ang II (Losartan, *P* = 0.045; 20 mg/L Lignans, *P* = 0.01; others, *P* < 0.001). The experiment demonstrated that *Eucommia* lignans could suppress Ang II-induced AR expression in RMCs.

## Discussion

*Eucommia* lignans (10 to 90 mg/L) was incubated with RMCs, according to our previous study with renal tubular epithelial cells (HK-2 cells) [12]. *Eucommia* lignans at 90 mg/L affected the normal growth of RMCs. Therefore, *Eucommia* lignans amounts in the subsequent experiments were set as 20, 40 and 80 mg/L.

The result consistent with those previous reports on the pathogenesis of hypertensive glomerulosclerosis [3,5], and stimulates MC proliferation and biosynthesis of ECM including mainly Col IV, fibronectin, Col I and Col III in *in vivo* or *in vitro*[4,23-28]. Our present study found that Ang II (10 nM) stimulated proliferation and production of Col IV, fibronectin and Col I in RMCs, and that both mRNA and protein of Col III were over-expressed in RMCs induced by Ang II.

In the current study, Ang II-induced RMC proliferation was significantly inhibited by *Eucommia* lignans, and there was a reduction in the raised expression of Col I, Col III, Col IV and fibronectin at both mRNA and protein levels. However, the mechanisms of *Eucommia* lignans in preventing Ang II-induced proliferation of RMC and production of ECM are poorly defined. According to some reports, AR, as a member of the aldo-ketoreductase superfamily, is involved in the cellular proliferation and ECM (Col I, Col IV and fibronectin) production induced by TGF-β1 or PDGF in human or rat MCs, and TGF-β1 and PDGF are downstream genes of Ang II [13-16]. Therefore, we tested the hypothesis that AR might participate in the pathological process in RMCs induced by Ang-II. This study demonstrated both AR mRNA and protein levels increase in RMCs were induced by Ang-II, in addition to our previous finding that *Eucommia* lignans decreased the production of Col III by degrading the expression of AR protein in SHR renal tissue [12], showed that the *Eucommia* lignans’ effects on Ang II-induced pathological changes in RMCs involved the reduction in the expression of AR. Our further studies will examine the signal pathways for the reduction of *Eucommia* lignans by AR expression.

## Conclusions

*Eucommia* lignans (*Du-zhong*) inhibited Ang II-stimulated extracellular matrix biosynthesis in mesangial cells.

## Abbreviations

Ang II: Angiotensin II; AR: Aldose reductase; Col I: Collagen type I; Col III: Collagen type III; Col IV: Collagen type IV; ECM: Extracellular matrix; GAPDH: Glyceraldehyde phosphate dehydrogenase; MCs: Mesangial cells; MTT: 3-(4, 5-dimethylthiazol-2-yl)-2, 5-diphenyl tetrazolium bromide; NCS: Newborn calf serum; PDGF: Platelet-derived growth factor; RAS: Rennin-angiotensin system; SHR: Spontaneous hypertensive rat; TGF-β1: Transforming growth factor-β1; TNF-α: tumor necrosis factor-α.

## Competing interests

The authors declare that they have no competing interests.

## Authors' contributions

DO and HZ designed this study. ZL, XD, LL, HL, XJ, YT, and PL performed the experiments. ZL, XD and WH wrote the manuscript. All authors have read and approved the final manuscript.
